# The Q705K Polymorphism in NLRP3 Is a Gain-of-Function Alteration Leading to Excessive Interleukin-1β and IL-18 Production

**DOI:** 10.1371/journal.pone.0034977

**Published:** 2012-04-17

**Authors:** Deepti Verma, Eva Särndahl, Henrik Andersson, Per Eriksson, Mats Fredrikson, Jan-Ingvar Jönsson, Maria Lerm, Peter Söderkvist

**Affiliations:** 1 Division of Cell Biology, Department of Clinical and Experimental Medicine, Faculty of Health Sciences, Linköping University, Linköping, Sweden; 2 Department of Clinical Medicine, School of Health and Medical Sciences, Örebro University, Örebro, Sweden; 3 Division of Microbiology and Molecular Medicine, Department of Clinical and Experimental Medicine, Faculty of Health Sciences, Linköping University, Linköping, Sweden; 4 Division of Rheumatology, Department of Clinical and Experimental Medicine, Faculty of Health Sciences, Linköping University, Linköping, Sweden; 5 Division of Occupational and Environmental Medicine, Department of Clinical and Experimental Medicine, Faculty of Health Sciences, Linköping University, Linköping, Sweden; University of Bonn - Institute of Experimental Hematology and Transfusion Medicine, Germany

## Abstract

**Background:**

The Q705K polymorphism in NLRP3 has been implicated in several chronic inflammatory diseases. In this study we determine the functional role of this commonly occurring polymorphism using an *in-vitro* system.

**Principal Findings:**

NLRP3-WT and NLRP3-Q705K were retrovirally transduced into the human monocytic cell line THP-1, followed by the assessment of IL-1β and IL-18 levels in the cell culture supernatant. THP-1 cells expressing the above NLRP3 variants were sorted based upon Green Fluorescent Protein (GFP) expression. Cytokine response to alum (one of the most widely used adjuvants in vaccines) in the cells stably expressing NLRP3-WT and NLRP3-Q705K were determined. IL-1β and IL-18 levels were found to be elevated in THP-1 cells transduced with NLRP3-Q705K compared to the NLRP3-WT. Upon exposure to alum, THP-1 cells stably expressing NLRP3-Q705K displayed an increased release of IL-1β, IL-18 and TNF-α, in a caspase-1 and IL-1 receptor-dependent manner.

**Conclusions:**

Collectively, these findings show that the Q705K polymorphism in NLRP3 is a gain-of-function alteration leading to an overactive NLRP3 inflammasome. The option of IL-1β blockade may be considered in patients with chronic inflammatory disorders that are unresponsive to conventional treatments.

## Introduction

Inflammasomes are essential regulators of interleukin (IL)-1β production. Upon activation, NLRP3 (formerly known as Cryopyrin/CIAS1/NALP3), associates with ASC (PYCARD) adaptor and pro-caspase-1 to form the NLRP3 inflammasome. This interaction leads to the activation of caspase-1, which proteolytically processes pro-IL-1β and pro-IL-18 to form active IL-1β and IL-18 [1]. CARD-8 has been suggested to be a binding partner of the inflammasome [2] but its role in the inflammasome is still a matter of debate. Gain-of-function mutations in the gene encoding NLRP3 can lead to its constitutive activation resulting in an uncontrolled IL-1β production. NLRP3 mutations have been implicated in hereditary inflammatory diseases and are grouped under cryopyrin-associated periodic syndromes (CAPS) or cryopyrinopathies [3]. The CAPS are regarded as monogenic disorders, comprising a trio of autoinflammatory conditions varying in severity of disease manifestation: familial cold associated syndrome (FCAS) being the mildest form, Muckle-Wells syndrome (MWS) being intermediate, and neonatal onset multisystem disorder (NOMID, also known as chronic infantile neurological cutaneous and articular syndrome; CINCA), being the most severe. Patients suffering from these syndromes typically present with fever, skin rashes and arthritis-like symptoms. IL-1β plays a central role in the pathogenesis of these disorders, which is proved by the remarkable improvement in symptoms upon IL-1β blockade [4].

We previously reported a patient with chronic inflammatory symptoms carrying the gene polymorphisms Q705K in NLRP3 (reported in the infevers database as Q703K (http://fmf.igh.cnrs.fr/ISSAID/infevers/) and C10X in CARD-8 [6]. This patient had a long history of arthritis and antibiotic-resistant fever but lacked the typical signs of FCAS, MWS or NOMID. Remarkably, like in other typical CAPS patients, IL-1 receptor (IL-1R) blockade using anakinra effectively abolished the patient's symptoms. The abundance of this polymorphism in the general population (5–11%) [5] makes it highly relevant to study its functional significance, particularly since several studies have shown a correlation of Q705K alone or in conjunction with C10X with increased risk of chronic inflammation [7], [8], [9], [10], [11]. Our results reveal a gain-of-function phenotype of the Q705K polymorphism which, unlike the other known genetic alterations in NLRP3, is associated with only moderately increased IL-1β levels. These findings combined with above epidemiological data are indicative of an important role of this polymorphism in susceptibility to chronic inflammatory conditions. Our findings also provide insight into the requirement of effective IL-1R activation for efficient IL-1β production in cells with overactive inflammasomes, demonstrating an autocrine feedback loop for IL-1β release under sterile conditions.

## Results

### Enhanced IL-1β and IL-18 release in THP-1 cells retrovirally transduced with NLRP3-Q705K

**Figure 1:**
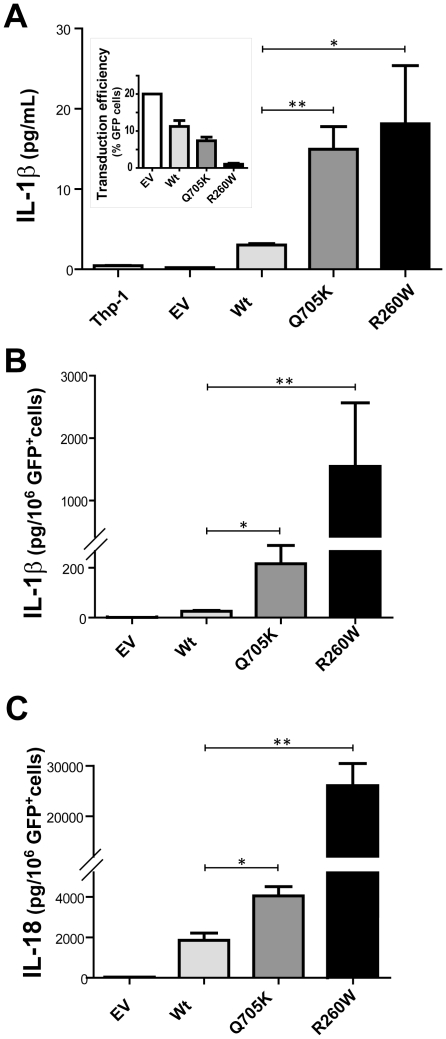
Basal levels of cytokine production by NLRP3-tranduced THP-1 cells. THP-1 cells were transduced with NLRP3-Q705K, NLRP3-R260W, NLRP-3 WT or Empty Vector (EV, *i.e.* no NLRP3) all including the gene for GFP. ***A***, IL-1β secretion was determined 48 h after transduction using ELISA, ***Inset***, Retroviral transduction efficiency was determined by monitoring GFP expression using flow cytometry. ***B***, IL-1β determined in A was corrected for the number of GFP+ cells in culture. ***C***, IL-18 secretion was determined 48 h after transduction and corrected for GFP+ cells in culture. ***A–C***, Data represents one of three independent experiments. Experiment was performed in triplicates and represented as Mean ± SEM.

To determine whether the Q705K variant of NLRP3 led to a spontaneous cytokine production, the cells were transduced with a retroviral vector expressing NLRP3-wild type (WT) or NLRP3-Q705K. The MWS-associated mutation NLRP3-R260W was used as a positive control and the empty vector (EV) was used as negative control. IL-1β, IL-18 and TNF-α levels were measured 48 h after retroviral transduction. Cells expressing NLRP3-Q705K demonstrated a five-fold increase in IL-1β levels as compared to the WT control, indicating that this variant leads to a constitutively activated inflammasome (Fig. 1*A*). The NLRP3-R260W variant displayed a seven-fold increase as compared to the WT control. The use of the caspase-1 inhibitor Z-YVAD-FMK with NLRP3-Q705K-expressing cells during the 48 hour time period resulted in IL-1β levels reduced to 47% (data not shown) demonstrating that this process is to a large extent dependent on caspase-1. The transduced cells were expressing GFP, the fluorescence of which was used to determine the transduction efficiency using flow cytometry (Fig. 1*A* *inset*). Correction of the IL-1β levels for the number of GFP-positive cells rendered data showing a similar trend, with the Q705K variant inducing higher levels of IL-1β than the NLRP3- WT (Fig. 1*B*). IL-18 levels were also found to be elevated in samples transduced with NLRP3-Q705K and NLRP3-R260W (Fig. 1*C*; GFP-corrected data). The TNF-α levels in all samples were below the detection level at this early time point after transduction.

### NLRP3-Q705K is a gain-of-function alteration

**Figure 2:**
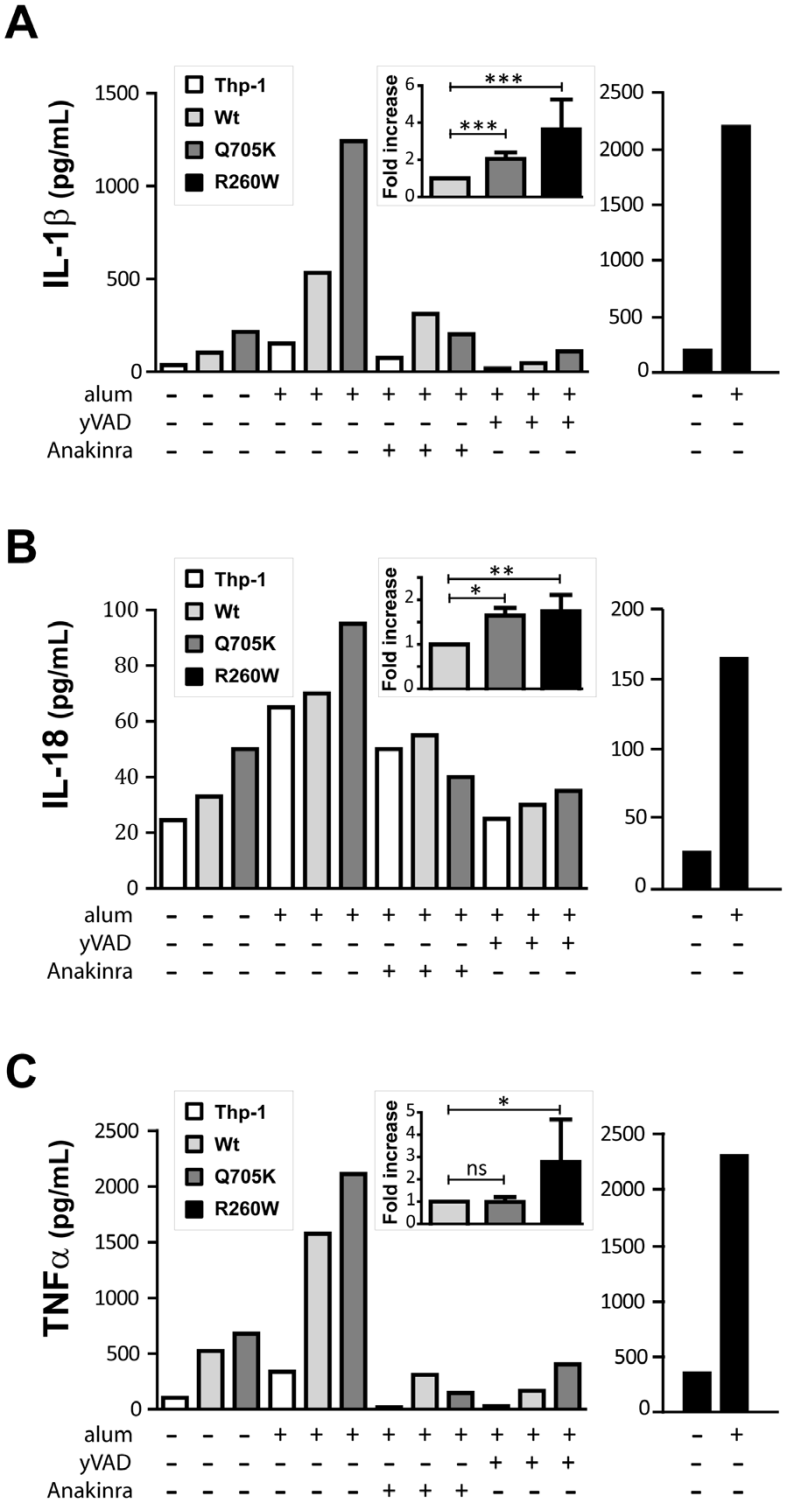
Alum-induced levels of cytokine production by NLRP3-tranduced THP-1 cells. THP-1 cells expressing NLRP3-Q705K, NLRP3-R260W, NLRP3-WT were pre-treated with Z-YVAD-FMK (50 µM, 2 h) or anakinra (5 µg/mL, 1 h) and exposed to alum (130 µg/mL, 4 h). ***A***, IL-1β, ***B***, IL-18 and ***C***, TNF-α secretion levels in unstimulated, alum, Z-YVAD-FMK or anakinra treated THP-1 cells are shown. Data represents one independent experiment, out of 2–5. ***Inset***, Fold higher ***A***, IL-1β, ***B***, IL-18 and ***C***, TNF-α in NLRP3-Q705K and NLRP3-R260W compared to NLRP3- WT. Data represent mean ± SEM of 4–5 independent experiments.

To obtain a population of cells stably expressing the wild type and mutant variants of NLRP3, we sorted the THP-1 cells expressing GFP and expanded them in culture medium. Next, in order to determine the production of cytokines in resting and stimulated THP-1 expressing NLRP3-Q705K, PMA-differentiated cells were stimulated with alum, which is one of the most widely used adjuvants in vaccines [12] and known to trigger sterile inflammation through the NLRP3 inflammasome [13], [14], [15]. Alum exposure resulted in a substantial increase in IL-1β production (Fig. 2*A*) in THP-1 expressing NLRP3-WT and the two mutant variants, the latter ones giving a more pronounced response (Fig. 2*A*). The NLRP3-Q705K displayed a statistically significant two-fold increase compared to WT expressing cells (Fig. 2*A* inset). A similar trend was observed in IL-18 levels upon alum stimulation, where NLRP3-Q705K and -R260W-expressing THP-1 cells showed higher levels compared to the WT-expressing THP-1 cells (Fig. 2*B and 2B* inset). This demonstrates that the Q705K variant of NLRP3 leads to an enhanced basal and stimulated inflammatory response and thus is a gain-of-function alteration resulting in a more pronounced cytokine production under sterile conditions, both in unchallenged THP-1 cells and in response to alum.

### The alum-induced IL-1β and IL-18 release by Q705K is caspase-1 and IL-1R-dependent

To confirm the specificity of our system, we tested whether the IL-1β and IL-18 production was dependent on caspase-1, which has been shown to be crucial for production of the studied cytokines in recent reports [13], [14]. Figure 2 shows a remarkable decrease in cytokine levels upon treatment with the caspase-1- inhibitor Z-YVAD-FMK, indicating that alum induces a caspase-1-dependent IL-1β and IL-18 release. It is well established that TLR ligands enhance the production of pro-IL-1β via NF-κB activation, and such microbial ligands are therefore widely used in *in vitro* studies to enhance IL-1β secretion. Given the fact that TLRs and the IL-1R share the same signalling pathway for NF-κB activation [16], [17], we investigated to what extent the IL-1R was required for the observed cytokine production using anakinra in our sterile setting. Anakinra is a recombinant IL-1R antagonist, which competitively binds to the IL-1R and blocks downstream IL-1β signalling [18]. As shown in Figure 2, a substantial decrease in both IL-1β and IL-18 production was seen after anakinra treatment of both NLRP3-WT and -Q705K-expressing THP-1 cells showing that the production of both cytokines in response to a sterile stimulus like alum involves an autocrine positive feedback mechanism via the IL-1R.

**Figure 3:**
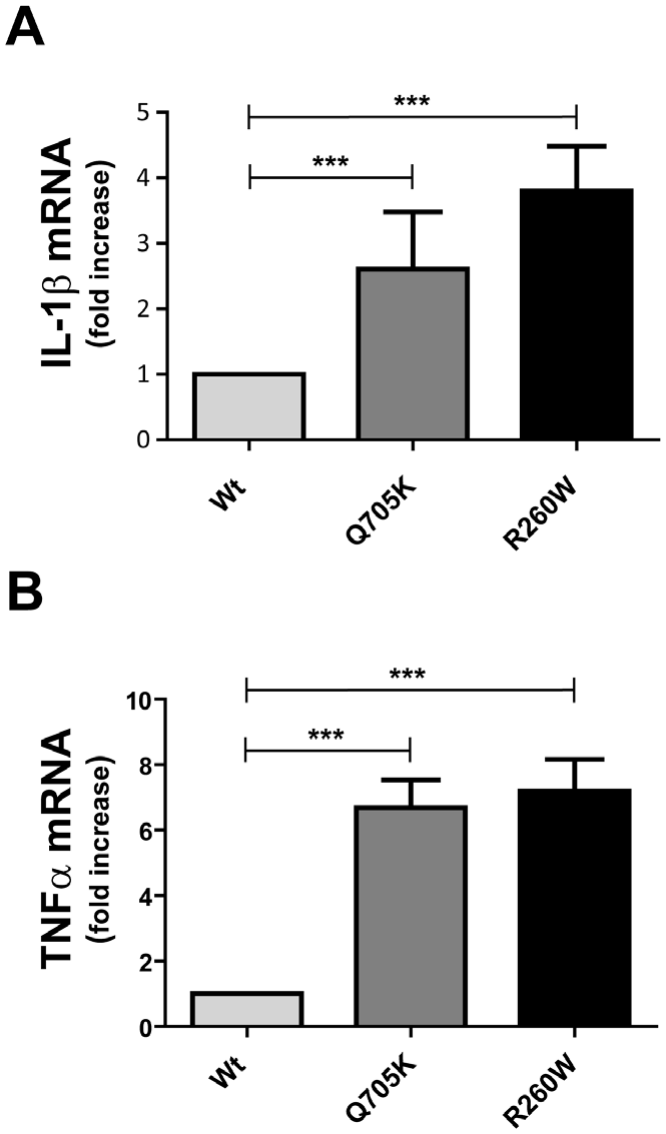
Basal levels of mRNA expression of pro-IL-1β and TNF-α genes represented as folds increase over NLRP3-WT. Data represents Mean ± SEM of 3 independent experiments performed in triplicates.

Generally upregulated cytokine levels in patients with mutated NLRP3 have been shown [19] and in line with this observation we could detect elevated TNF-α levels in THP-1 cells stably expressing WT and mutant NLRP3 (Fig. 2*C*). A substantial increase in secreted TNF-α levels in alum-stimulated transduced samples could be observed, however, there were no significant difference between alum-stimulated NLRP3-WT- and -Q705K-expressing THP-1 cells (n = 4) (Fig. 2*C* inset). Our observation that a substantial decrease in TNF-α levels was obtained with Z-YVAD-FMK and anakinra treatment suggests that the release of IL-1β triggers TNF-α release via binding to the IL-1R. These two cytokines have previously been shown to correlate with each other [20], [21], [22] but the underlying mechanism is not clearly understood. To corroborate our findings made with the ELISA assays, we performed a quantitative real-time PCR. As expected, the mRNA levels of pro-IL-1β were found to be significantly higher in NLRP3-Q705K as compared to NLRP3-WT (Figure 3*A*). TNF-α mRNA levels were also significantly increased in the NLRP3-Q705K (Figure 3*B*). These data suggest that the NLRP3-Q705K, likely through enhanced secretion of IL-1β, up-regulates expression levels of both pro-IL-1β and TNF-α. In order to determine if the enhanced expression levels are regulated by IL-1R, we used anakinra. However, the expression levels of these genes were not affected by anakinra treatment (data not shown), suggesting that the mechanism behind transcriptional regulation of IL-1β and TNF-α must be different from the IL-1R regulated release of these cytokines.

## Discussion

In our previous report of a patient with recurrent inflammatory symptoms, Q705K in NLRP3 and C10X in CARD-8 were found to coincide with increased caspase-1 activity and IL-1β secretion [6]. Our and others' earlier published epidemiological data obtained from studies of patients with rheumatoid arthritis and Crohn's disease pointed towards a role for this polymorphism in susceptibility to chronic inflammatory conditions [8], [9], [10].

We undertook these studies with the aim of evaluating the role of Q705K in IL-1β production. The human monocytic cell line THP-1 was used, since it expresses all the components of the NLRP3 inflammasome [23], [24] and does not carry the NLRP3-Q705K alteration. In addition, this cell line is heterozygous for the C10X in CARD-8, which makes it a suitable model to study the effect of Q705K with a heterozygous C10X background. The increased IL-1β and IL-18 cytokine levels obtained upon retroviral expression of the Q705K into THP-1 cells indicate that it is a gain-of-function variation leading to an overactive inflammasome. The moderately produced IL-1β levels from NLRP3-Q705K (compared to the MWS-causing NLRP3-R260W) suggest an important role of this common variant in chronic inflammatory diseases. In line with previous reports, we observed lower transduction efficiencies in both the mutant variants, reflecting the induction of inflammasome-linked cell death [24], [25], [26]. However, since the caspase-1 inhibitor Z-YVAD-FMK effectively reduced IL-1β levels, we can rule out the possibility that the produced IL-1β was a result of pyronecrosis, which previously has been shown to be caspase-1 independent [26]. Although using the same cell line and constructs as in a previous report [24], we observed lower IL-1β levels, which can likely be attributed to lab variations in handling of cells and methods. CARD-8 has been suggested to be a binding partner of NLRP3 but its functional role for inflammasome function is not clear. A recent study using THP-1 cells showed that knocking-down CARD-8 did not affect IL-1β production in response to influenza virus [27]. However, given the inhibitory role of CARD-8 in the regulation of NF-κB [28], it is possible that the lower amounts of functional CARD-8, as would be the case with the truncating C10X mutation, results in a more pronounced effect of any activating NLRP3 mutation. The relative contribution of CARD-8-C10X to inflammatory disorders in association with inflammasome mutations requires thorough investigation and will hence be the subject of a separate study.

As a trigger for sterile inflammation we used alum, which is one of the most widely used vaccine adjuvants and a known activator of the NLRP3 inflammasome, to investigate the release of cytokines from THP-1 cells expressing wild type and mutated NLRP3 [13], [15]. Indeed, we found that mutated NLRP3 produced more cytokines in response to alum as compared to wild type NLRP3. These findings suggest that the NLRP3-Q705K variant is associated with a lower threshold for inflammasome activation, potentially implying an increased genetic susceptibility for inflammatory stimuli in individuals possessing this variant. Further confirmation of NLRP3-Q705K being a gain-of-function variant was obtained when elevated spontaneous mRNA levels of pro-IL-1β were detected in the THP-1 cells stably expressing this variant. In agreement with a previous report suggesting TNF-α to be a late event associated with IL-1β release [2], we could detect TNF-α in cells stably expressing the mutant variants but not in the freshly transduced cells. The increased TNF-α release was likely a downstream effect of IL-1β release, since anakinra could block the effect. The lack of significantly increased TNF-α release in the NLRP3-Q705K compared to the WT expressing cells could possibly be due to sub-threshold levels of IL-1β observed in this polymorphic variant compared to the NLRP3-R260W. How NLRP3-Q705K enhances IL-1β and TNF-α expression independently of the IL-1R remains elusive, but the involvement of other pathways cannot be ruled out. For instance, signalling through IL-18R has been shown to activate NF-κB [29], which could lead to increased expression of pro-IL-1β and TNF-α [29], [30], [31]. Alternatively, apoptotic speck-like protein (ASC), which is another component of the inflammasome, might through activation by the NLRP3-Q705K directly up-regulate NF-κB-regulated genes such as pro-IL-1β and TNF-α independently of the IL-1R [32], [33], [34], [35].

A number of patients displaying either classical or atypical CAPS, possessing NLRP3-Q705K alone or in combination with other polymorphisms has previously been reported [4], [6], [36], [37] many of which have successfully been treated with anakinra [4], [6], [25], [37]. However, as all these patients do not fit into the conventional CAPS phenotype, the interpretation of NLRP3-Q705K presents a diagnostic challenge to the clinicians. Nonetheless, IL-1β blockade has been successful in treating many of these patients [4], [6], [25], [37]. In a recent paper we have reported four patients with inflammatory symptoms carrying the NLRP3-Q705K in combination with CARD-8-C10X, where increased IL-1β release from patient's monocytes was observed [38].

Based upon our *in vitro* studies as well as earlier published epidemiological data [8], [9], [10], [11], we suggest that these polymorphisms, in conjunction with an environmental cue such as an infection, or with other, yet unidentified genetic variations, predispose for enhanced inflammation. Using a similar THP-1 cell model, we have earlier published data showing a novel M299V mutation in NLRP3 to be functional [25]. The contribution of environmental factors rather than additional genetic alterations would explain the later-onset of symptoms in some of the patients, compared to the neonatal to early onset in most of the CAPS patients carrying the severe disease-causing mutations [4]. A similar situation has been described in FMF where the alteration MEFV-E148Q, referred to as a polymorphism due to its presence in 10% of asymptomatic individuals, is detected in patients with milder symptoms [39]. In this case, environmental factors are suggested as the responsible triggers of inflammation [40], [41]. Increasing numbers of studies showing synergistic effects between polymorphisms in different genes associated to autoinflammation are emerging [36], [42], [43], signifying the need of a careful diagnosis of the patients possessing low-penetrance alterations.

In many reports, TLR ligands are used to drive the production of pro-IL-1β for efficient IL-1β release [14], [44], [45]. Here we studied the response of the inflammasome under sterile conditions, where no TLR ligands or other bacterial products were used to enhance transcription of pro-IL-1β. Our study reinforces the importance of IL-1β signalling through its receptor for effective production of the same cytokine under sterile conditions, which has previously been demonstrated in healthy individuals [46] as well as in CAPS patients [47], [48]. We also show that IL-1β release precedes TNF-α release via binding to the IL-1R, and that treatment with anakinra decreases both IL-1β and TNF- α releases, which has earlier been shown to occur *in vivo* in mice [49]. It is possible that such a pro-inflammatory feedback loop, once established through different exogenous and endogenous stimuli, may be difficult to break in patients with alterations in the NLRP3 inflammasome.

The discovery of disease-causing mutations in NLRP3 has led to the recognition of a connection between autoinflammatory disorders and a dysregulated innate immunity. The molecular mechanism for the constitutive phenotype of disease-causing NLRP3 alterations is not known, but it is suggested that this is due to structural instability leading to unprovoked association of NLRP3 with its adaptor molecules, causing spontaneous IL-1β production [4], [50]. Our present data are in agreement with previous results showing missense alterations in exon 3 of NLRP3 to be associated with increased IL-1β [23].

In summary, we report an increased activity of the NLRP3-Q705K polymorphism as demonstrated by the increased spontaneous and stimulated release of cytokines under sterile conditions. Our study points to the need of extending the conventional categorization of patients with non-classical CAPS, at the same time the risk for over-interpretation of this genotype can be avoided with careful diagnosis. The option of IL-1 blockade might be considered, particularly in the patients unresponsive to standard treatments.

## Materials and Methods

### Site-Directed Mutagenesis

Mutant forms of NLRP3 (NM_004895.3) were generated by site-specific mutagenesis of the wild type NLRP3 cloned into a retroviral vector, pHSPG, tagged with Green Fluorescent Protein (GFP), using the protocol of Quikchange II. The following primer pairs were used:

Q705K, Forward (F) - gacaccttgatatggtgaagtgtgtcctcccaagc & Q705K, Reverse (R) – gcttgggaggacacacttcaccatatcaaggtgtc R260W, (F) - ctatctgttctatatccactgttgggaggtgagccttgtgacacag & R260W, (R) - ctgtgtcacaaggctcacctcccaacagtggatatagaacagatag

### Genotyping

THP-1 cells (American Type Culture Collection, Rockville, MD) were genotyped for Q705K and C10X alterations using Taqman assays (C_25648615_10 and C_551339_10, respectively) following the manufacturers protocol (Applied Biosystems, Carlsbad, CA).

### Cell lines

293T cells (American Type Culture Collection, Rockville, MD) were cultured in DMEM with 10% FBS and penicillin/streptomycin. THP-1 cells were cultured in RPMI 1640 medium supplemented with 10% heat-inactivated fetal bovine serum (FBS) and penicillin/streptomycin. The cells were maintained in a humidified atmosphere of 5% CO_2_ at 37°C and passaged every third day.

### Retroviral Transductions

For packaging of virus particles, 293T cells were transfected with plasmids encoding WT and mutant NLRP3, pGag-Pol and pVSV-G encoding plasmids as described by Eliasson [51]. 48 h after transfection, viral supernatants were collected. Viral supernatants were titrated on 293T cells and in all the cases were found to be higher than 1×10^6^ particles/ml. THP-1 cells (0.5×10^6^) were transduced by suspending in one volume cell culture medium plus one volume virus supernatant, after which polybrene (Sigma-Aldrich,St. Louis, MO) was added at a final concentration of 4 µg/mL. The virus/cell mixture was centrifuged at 1500×g for 1.5 h at room temperature. The supernatant was replaced with fresh medium. After 48 h, the cell supernatant was harvested and stored at −70° C for cytokine analysis. The cells were subjected to flow cytometry on a FACScan (BD Biosciences, Franklin Lakes, NJ) to determine the efficiency of transduction based on GFP expression. GFP-positive THP-1 cells were sorted by FACS, expanded under standard culture conditions and used for the cell stimulation assays.

### Blockade of caspase-1 and IL-1

To investigate the role of caspase-1 in Q705K induced IL-1 production, 50 µM caspase-1 inhibitor Z-YVAD-FMK (Cayman Chemicals, Ann Arbor, Michigan) was added to the cell culture medium during the 48 hour retroviral transduction or 2 h prior to addition of stimuli as indicated.

To more specifically assess the role of IL-1R blockade on cytokine production, the IL-1R antagonist anakinra (Kineret, Amgen, Thousand Oaks, CA) was added to a final concentration of 5 µg/mL in the incubation medium 1 hr before the addition of stimuli.

### Stimulations

THP-1 cells stably expressing WT and mutant forms of NLRP3 were grown to a density of 1.5×10^6^/mL in cell culture flasks. 24 h before stimulations, the cells were differentiated by treatment with 0.5 µM phorbol 12-myrsitate 13-acetate (PMA; Sigma-Aldrich) for 3 h. The cells were then washed with PBS and seeded at a density of of 1×10^6^ cells/well in 12-well plates in standard RPMI medium. The next day, cells were washed with PBS and 1 mL RPMI medium was added. Aluminium Hydrogel (Alum; Sigma-Aldrich) was used at 130 µg/mL for 4 h to stimulate the cells.

### Detection of cytokines

The cytokines IL-1β, IL-18 and TNF-α were assessed in the cell culture media using ELISA. The lower detection limit of IL-1β and IL-18 (R&D systems, Minneapolis, MN and MBL International) were 0.16 pg/mL and 12.5 pg/mL, respectively, while that for TNF-α (Abcam, Cambridge, MA) was 25 pg/mL.

### Gene Expression

Total RNA was isolated from THP-1 cells using TRIzol® (Invitrogen) and reverse transcribed to cDNA with Superscript II (Invitrogen) following the recommended protocol. mRNA expression of pro-IL-1β, *F*-cctgcgtgttgaaagatgat & *R*-actgggcagactcaaattcc and TNF-α *F*-cagagggcctgtacctcatc & *R*-gaggttgaccttggtctggt, were determined in triplicates using the SYBR green PCR kit on the 7900HT sequence detection system (Applied Biosystems, Carlsbad, CA), β-actin, *F*-acccagcacaatgaagatca & *R*-tcgtcatactcctgcttgct, was used for samples normalization and Ct values were calculated using the 2^−∧∧ct^ method.

### Statistical methods

Data are represented as mean ± SEM in figures 1, 2, 3. Statistical comparisons were performed by Student's unpaired t test (figure 1) or two-way ANOVA with Tukey's correction (figures 2) using the software package SPSS 18.0. * represents *P* values<0.05, ** represents *P* values<0.01 and *** represents *P* values<0.001.
